# Adverse Childhood Experiences and COVID-19 Stress on Changes in Mental Health among Young Adults

**DOI:** 10.3390/ijerph191912874

**Published:** 2022-10-08

**Authors:** Meshari A. Alradhi, Jessy Moore, Karen A. Patte, Deborah D. O’Leary, Terrance J. Wade

**Affiliations:** 1 Department of Health Sciences, Faculty of Applied Health Sciences, Brock University, 1812 Sir Isaac Brock Way, St. Catharines, ON L2S 3A1, Canada; 2 Brock-Niagara Centre for Health and Well-Being, 130 Lockhart Dr, St. Catharines, ON L2T 1W5, Canada

**Keywords:** adverse childhood experiences (ACEs), COVID-19, stress, mental health, young adults

## Abstract

The COVID-19 pandemic has been linked to poor mental health outcomes and may be particularly damaging for young adults who may be more affected by governmental pandemic responses such as mandatory school and work closures, online schooling, and social isolation. Exposure to Adverse Childhood Experiences (ACEs) has also been shown to have a significant impact on mental health among young adults. This prospective study examined whether young adults with higher ACE profiles were more vulnerable to COVID-19 stressors. Using pre-COVID-19 data from the Niagara Longitudinal Heart Study and a follow-up online survey during COVID-19, we examined 171 young adults and found that high COVID-19-related stress, especially emotional and relationship stress, led to a greater reduction in mental health among young adults with higher levels of ACEs. Findings indicate that young adults with high ACE profiles may benefit from resources and intervention programs directed at mental health in times of crisis, such as the COVID-19 pandemic.

## 1. Introduction

ACEs are generally defined as exposure to traumatic experiences such as abuse and maltreatment, severe household dysfunction, and witnessing intimate partner violence involving parents/caregivers before the age of 18 [1]. They have been identified as a significant risk factor for many adult health issues, including obesity, mental disorders, health-risk behaviors such as other substance use, and chronic diseases such as diabetes and cardiovascular disease [2,3,4,5,6]. Higher exposure to ACEs may also increase individuals’ vulnerability to subsequent stressors. That is, those with higher exposure to ACEs may be at greater risk of adverse health outcomes when exposed to subsequent individual- or population-level stressors, such as earthquakes and tornados, civil unrest and conflict, or global pandemics. These population-level events severely disrupt the daily routines of whole communities, threatening their livelihoods and even their lives [7]. The COVID-19 pandemic and associated government-related responses which included school and workplace closures, physical distancing measures, and mandatory isolation and quarantine, in addition to the health risks of COVID-19, have been shown to have negative mental health effects [8,9,10,11,12,13,14,15]. To our knowledge, there have been few studies that examined how ACEs may compound the stressors associated with the COVID-19 pandemic.

Young adults, as a cohort in a transitional life course stage, may be particularly affected by the COVID-19 pandemic and the governmental measures implemented to mitigate its impact [7,16,17,18,19]. First, mandated school and work closures, moving to online education, physical distancing, and quarantining have put constraints on the ability to cultivate friendships and long-term relationships, especially intimate partnerships, which are indicative of this age period [20]. This constraint in forming such relationships may affect mental and emotional health, as young adults have been shown to be more dependent on their social networks to cope with negative emotional responses than other adult cohorts [21]. Second, young adults are less likely to have secure, stable, full-time employment with benefits and sick-leave, and have fewer savings to endure work reductions and unemployment. In fact, those young adults of 15–24 years of age suffered the greatest job loss/work reduction in Canada during COVID-19, due mainly to their over-representation in the tourism, food services, and retail industries [22].

It is unclear whether past exposure to ACEs compounds the negative effect of COVID-19-related stressors, placing some individuals at even greater risk of negative mental health outcomes. A few studies have examined this connection linked to other population-level events and experiences. For example, one study examined the impact of ACEs on 549 Syrian child and adolescent refugees fleeing the civil war. Surprisingly, they found that exposure to ACEs was the most important predictor of PTSD, operating independently of their exposure to the war and refugee experience [23]. Another study that examined US veterans, who had served on active duty in the United States Armed Forces either during the draft era (prior to 1973) or during the all-volunteer era (post-1973), examined to what extent exposure to ACEs may have impacted the health behaviors of veterans. This study found that while ACEs are more prevalent in the all-volunteer era, a higher exposure to ACEs was associated with poorer mental and general health independently of the veterans’ service era [24]. As such, it is unclear whether exposure to ACEs may impact on mental health problems independently of population-level events or whether they may increase one’s vulnerability to subsequent stress exposures. While we are not equating the effects of war and active military service to those of the COVID-19 pandemic, and by extension other types of disasters such as earthquakes and hurricanes, these studies support further exploration into the role of ACEs as predictors of mental health outcomes when exposed to stress.

With respect to COVID-19, we are aware of some studies that have examined the potential link with ACEs. One cross-sectional study found that Chinese adolescents with higher exposure to cumulative ACEs such as neglect and family abuse were most at risk psychologically where COVID-19 lockdowns and social isolation were imposed [25]. A second cross-sectional study examined adults across the age spectrum and found that individuals reporting exposure to more types of child maltreatment (i.e., sexual abuse, physical abuse, emotional abuse, or neglect) reported a higher perceived threat from COVID-19 and higher anxiety levels [26]. A final cross-sectional study assessed ACEs and depressive symptomatology among adults (mean 40.1 years of age) both retrospectively prior to the pandemic and contemporaneously from May 2020 to July 2020 [27]. They found ACEs have a significant effect on increases in symptoms of depression but they did not examine the moderating effect of ACEs on COVID-19 stress. One prospective longitudinal study examining parent-reported, internalizing symptoms among young adolescents found significant increases in sadness and fear/worry and a significant decrease in positive affect [28]. Moreover, they found no interaction between ACEs with ethnicity or race on mental health changes over time but did not examine how ACEs may compound the effect of COVID-19 stress on these outcomes. Only one study examined how ACEs influence the relationship between negative pandemic-related events and changes in mental health in a prospective, longitudinal study design [29]. Among adults (mean age 30.3 years), the effect of ACEs on mental health and substance use outcomes was mediated by negative COVID-19 events but they identified only one interaction between ACEs and negative COVID-19 events over time on increased drug use. The current study adds to this work by examining a community sample of young adults across a range of COIVD-19-related stressors on various mental health outcomes. This prospective analysis assesses within-individual changes in mental health from pre-COVID-19 to during COVID-19 to assess the independent effects of both ACEs and COVID-19-related stressors as well as their interaction effect on changes in mental health over time.

## 2. Materials and Methods

### 2.1. Sample

Pre-COVID-19 data came from the ongoing Niagara Longitudinal Heart Study (NLHS) [30]. From March 2017 to mid-March 2020, prior to the university-wide shutdown of all human research due to COVID-19, the NLHS recruited 248 participants aged 18 years or older from the Niagara region. These participants were recruited due to their participation in three previous community-level, baseline studies almost a decade earlier examining the cardiovascular health of children. NLHS testing took up to 4 hours and included anthropomorphic measures, non-invasive cardiovascular assessments, biological specimen collection, and the completion of a detailed self-reported questionnaire.

The COVID-19 follow-up questionnaire was sent to all 248 participants having consented to be followed up for future research. They were contacted using email and social media and asked to complete an online survey using Qualtrics XM online survey software (Qualtrics, Provo, UT, USA). Of the 248 contacted, 171 participants completed the survey, a response rate of 69%. Data collection occurred over an approximately 2-month period between 27 July 2020, and 5 October 2020, when Ontario, and Canada more generally, were in-between COVID-19 waves and were loosening social restrictions. The NLHS and the COVID-19 survey both received Research Ethics Board approval (#18-288; #20-313).

### 2.2. Measures

The NLHS questionnaire covered a broad array of topics including measures of ACEs and mental health (i.e., depression, stress, anxiety, hostility) The COVID-19 survey included many of the same questions as the initial NLHS questionnaire to allow for longitudinal analysis. In addition, the online survey included questions about COVID-19-related stressors and the impact of the lockdowns (e.g., job loss, financial strain, social isolation).

#### 2.2.1. Adverse Childhood Experiences (ACEs)

ACEs were assessed (pre-COVID-19) using the Childhood Trust Events Survey v.2.0 (CTES 2.0)—a 26-item inventory adapted from the Traumatic Stress Survey [31] that screens for exposure to traumatic childhood events occurring prior to the age of 18 years [32]. For comparability, we included 14 items of the CTES 2.0 that mirrored the 8 ACE domains identified in the original ACE study [1]. These items focused on experiencing childhood maltreatment, including sexual (two items), physical (one item), and emotional abuse (two items), and severe household dysfunction, including witnessing domestic violence (two items), having someone in the household suffering from serious mental illness or suicidal ideation (two items), neglect due to a family member being addicted to drugs or alcohol (two items), or being incarcerated (one item), and an unexpected separation from a parent or death of a family member (two items). Any positive response on an item resulted in a positive coding for the specific domain. Previous studies have provided support for the retrospective self-reporting of ACEs in adulthood [33,34,35], including good test–retest reliability (Cohen’s kappa values of 0.6–0.7 over 1 year) when assessing childhood maltreatment [33]. Findings suggest that individuals are willing to report ACEs when they are in a safe, comfortable environment [34,35]. In accordance with the original ACE Study [1] and our groups’ previous work [36,37,38], a positive response for any domain item was coded as a positive response for the ACE domain which were then summed to create a scale ranging from 0 to a threshold value of ≥4 ACEs.

#### 2.2.2. COVID-19 Stressors

COVID-19 stressors were measured based on work by Lavoie and Bacon (2020) [39]. We examined twelve COVID-19-related stressors grouped into five general categories including emotional (lonely/isolated, irritable/frustrated/angry, suspicious/distrustful), lifestyle (less physically active, diet gotten worse), substance use (increased how often/much I use alcohol, recreational drugs), financial (job hours cut/lost income, unable to pay rent/mortgage, unable to pay for food), and relationship stressors (serious arguments / physical fights with people I live with). For example, the participants were asked about their experiences of the following: “Because of COVID… I have” felt lonely and isolated; been less physically active; had serious arguments with the people I live with; had my job hours cut/lost income. Participants answered each item using four response categories including “not at all” (1), “very little” (2), “somewhat” (3), and “to a great extent” (4) [39].

#### 2.2.3. Mental Health Outcomes

All outcomes were measured as continuous variables with higher scores indicating greater mental health problems. Depressive symptomatology was assessed using the Centre for Epidemiological Studies-Depression scale (CESD) by Radloff [40] consisting of 20 items. It has been shown to have excellent validity and reliability in both children and young adults [41]. Anxiety and hostility were assessed using the 10-item anxiety and 6-item hostility subscales of the Symptom Checklist 90-Revised (SCL-90-R), respectively, which have been demonstrated to be both valid and reliable [42,43]. Perceived stress was measured using the 14-item Perceived Stress Scale shown to be both valid and reliable [44].

### 2.3. Statistical Analysis

All analyses were adjusted for sex (male and female), age, and education status, all measured pre-COVID-19. The analysis proceeded in four steps including an attrition analysis, descriptive statistics for pre-COVID-19 and during COVID-19 data, correlational analysis of the during COVID-19 data, and longitudinal, mixed-model regression analyses to adjust for biased standard errors and intraclass correlation within participants. The regression analyses assessed (1) the independent effect of ACEs and each COVID-19 stressor on change over time for each outcome and (2) the interaction between ACEs and each COVID-19 stress over time to assess their conditional effects on change over time for each outcome. All analyses were conducted using SAS 9.4 (SAS Institute, Cary, NC, USA).

## 3. Results

### 3.1. Attrition Analysis

The attrition analysis comparing NLHS (pre-COVID-19) data between those who participated in the during COVID-19 survey (171) to those who did not (77) found no differences across any of the mental health outcomes (results not shown). There were also no significant differences across sex or age. Those who participated reported significantly higher education status compared to those who did not complete the online survey. Descriptive statistics of the 171 participants are reported in Table 1. While overall mental health problems increased across all measures from pre- to during COVID-19, increases were only statistically significant for anxiety and hostility.

### 3.2. Correlation Analysis

The correlation analysis found that ACEs were significantly and positively correlated with all mental health outcomes measured during COVID-19. ACEs were also significantly correlated with frustration, drug use, inability to pay rent/mortgage and food, and serious arguments with the people one lives with. All mental health outcomes were significantly correlated with all emotional-based stressors, inability to pay for food, and serious arguments. In addition, alcohol use was correlated with anxiety while recreational drug use was correlated with depression, anxiety, and perceived stress. Decreased income was correlated with depression while inability to pay rent/mortgage was correlated with depression, hostility, and perceived stress (Table 2).

### 3.3. Mixed Effects Regression Analysis

Mixed effects, longitudinal regression assessed how exposure to ACEs and COVID-19-related stress related to changes across mental health outcomes employing a series of two-way and three-way interaction models. These models assessed the independent and conditional effects of ACEs with COVID-19 stressors over time (Table 3; Appendix A, Table A1 and Table A2). The first set of regression models assessed the independent effects of both the COVID-19 stressors and ACEs over time (separate two-way interactions with time) on changes in mental health outcomes, adjusting for age, sex, and education status. ACEs independently predicted changes in depression, adjusting separately for loneliness, frustration, income loss, inability to pay rent/mortgage and food, and serious arguments (Table A1). Interestingly, however, after adjusting for all COVID-19 by time interactions, the ACE by time interaction (change over time in mental health outcome) were negative. While these findings appear to be opposite to expectations, they do not identify a negative association but a convergence between the high and low ACE groups after adjusting for COVID-19 stress. ACEs were not related to any other outcome after adjusting for any COVID-19 stressor. The COVID-19 stressors that affected changes in depression independently of ACEs included emotional (suspiciousness), financial (reduced income and inability to pay for food), and relationship stressors (serious arguments). Increased hostility was associated with all emotional stressors, all lifestyle stressors (inactivity and poor diet), all financial stressors, and serious arguments after adjusting for ACEs. Finally, increases in perceived stress were associated with all emotional stressors, most financial stressors (reduced income, inability to pay rent/mortgage), and serious arguments.

**Figure 1:**
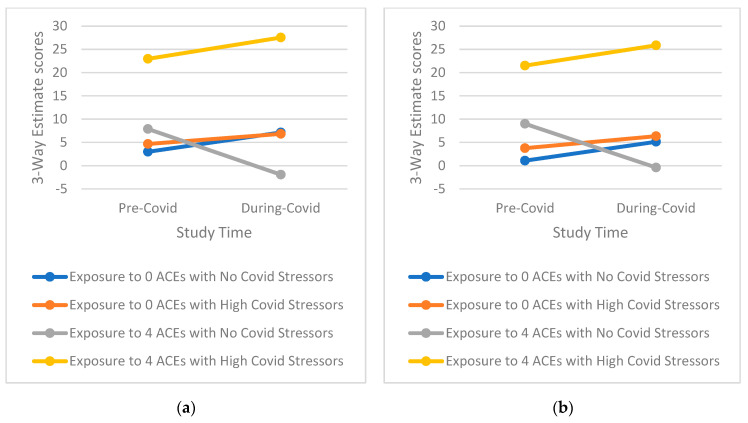
(**a**) Interaction of greater exposure to ACEs and high levels of Loneliness on Changes in Depressive Symptomatology among Young Adults from Before to During the COVID-19 Pandemic; (**b**) Interaction of greater exposure to ACEs and high levels of Frustration on Changes in Depressive Symptomatology among Young Adults from Before to During the COVID-19 Pandemic.

**Figure 2:**
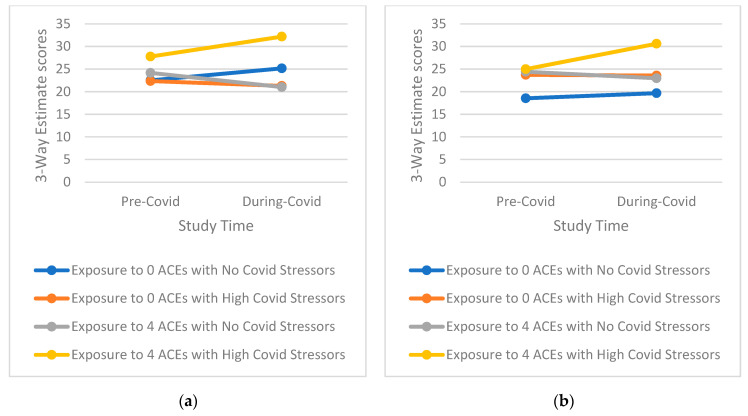
(**a**) Interaction of greater exposure to ACEs and high levels of Loneliness on Changes in Anxiety Symptomatology among Young Adults from Before to During the COVID-19 Pandemic; (**b**) Interaction of greater exposure to ACEs and high levels of Suspiciousness on Changes in Anxiety Symptomatology among Young Adults from Before to During the COVID-19 Pandemic.

**Figure 3:**
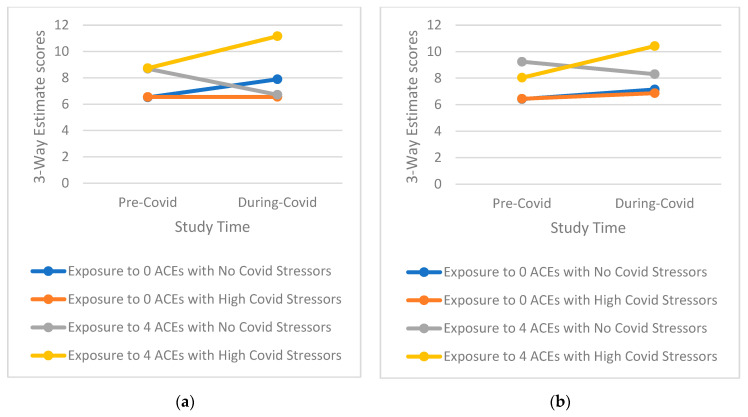
(**a**) Interaction of greater exposure to ACEs and high levels of Loneliness on Changes in Hostility Symptomatology among Young Adults from Before to During the COVID-19 Pandemic; (**b**) Interaction of greater exposure to ACEs and low levels of Physical Activity on Changes in Hostility Symptomatology among Young Adults from Before to During the COVID-19 Pandemic.

**Figure 4:**
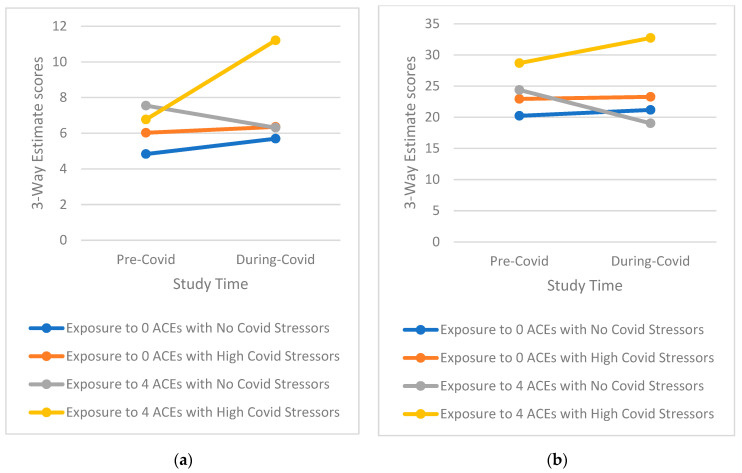
(**a**) Interaction of greater exposure to ACEs and high levels of Serious Arguments with a Roommates on Changes in Hostility Symptomatology among Young Adults from Before to During the COVID-19 Pandemic; (**b**) Interaction of greater exposure to ACEs and high levels of Frustration on Changes in Perceived Stress Symptomatology among Young Adults from Before to During the COVID-19 Pandemic.

**Figure 5:**
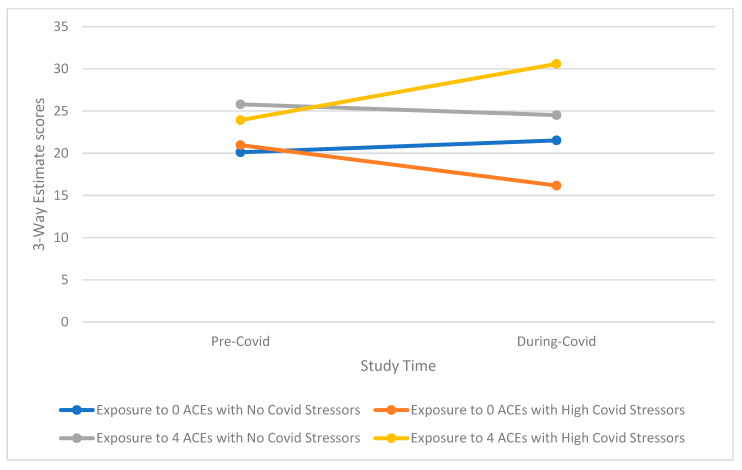
Interaction of greater exposure to ACEs and increased levels of Alcohol Consumption on Changes in Perceived Stress Symptomatology among Young Adults from Before to During the COVID-19 Pandemic.

Tests of three-way interactions exploring whether the effect of higher COVID-19 stress is conditional on the level of ACEs score on mental health outcomes over time indicated a notable pattern (Table 3, Appendix A, Table A2). This pattern shows that those with a high ACE profile and high reported COVD-19 stress, especially for emotional-type stress, have significantly greater increases in mental health symptomatology than those with either a high ACE profile or high reported COVID-19 stress as well as those with low ACEs and low reported COVID-19 stress (examples of three-way interactions comparing groups with 0 ACEs and ≥4 ACEs only for simplicity are presented in Figure 1, Figure 2, Figure 3, Figure 4 and Figure 5). Specifically, among those with a higher ACE profile, higher reported loneliness was associated with all outcomes, frustration was associated with depression, hostility, and perceived stress, and suspiciousness was associated with increased anxiety and perceived stress. Interestingly, increased hostility was associated with several COVID-19 stressors including lifestyle (physical inactivity and poor diet), financial (decreased income, inability to buy food), and relationship (having serious arguments with the people one lives with) stressors. For young adults exposed to a higher number of ACEs, increased anxiety was also associated with serious arguments with people living with those young adults. Finally, substance use (specifically increase in alcohol use) was linked to greater increases in perceived stress among those reporting more ACEs while increased recreational drug use was not associated with any outcome.

## 4. Discussion

This study examined how exposure to greater numbers of ACEs may compound the negative effects of COVID-19 stressors on changes in mental health among young adults. To our knowledge, it is one of the few studies that examines pre- to during COVID-19 changes in mental health prospectively based on ACEs exposure. The original NLHS study focused on young adults, providing a unique opportunity to study a cohort which may be especially vulnerable to the governmental responses to mitigating the impact of the COVID-19 pandemic based on their transitional life-course stage [45]. Specifically, young adults are more likely than older adults to be dependent on their social networks to cope with negative emotional responses [21] which are threatened by lockdowns, job losses, limits on social gatherings, the movement to online education, and limited opportunities to initiate and foster long-term, intimate relationships [46]. As well, emerging adults are less likely than older adults to have secure, stable, full-time employment with benefits and sick leave because they are more likely to work in retail and hospitality service sectors. Those employed in these sectors were more prone to job losses and reduced hours resulting from COVID-19 [22]. As these sectors generally have lower wages, they are less likely to have built sufficient savings to endure work reductions and unemployment.

Past research has made it clear that both ACEs and COVID-19 stress impact health and well-being. High exposure to ACEs has been independently linked to many mental health problems such as depression, substance use, antisocial behaviors, and personality disorders, as well as the risk of developing chronic physical conditions and diseases later in life [27,28,29,47]. The question addressed here, however, is whether exposure to a greater number of ACEs compounds the negative effects of COVID-19-related stressors on mental health among young adults. That is, are those who experience higher levels of ACEs more vulnerable to stressors related with COVID-19? Contrary to previous studies of early adolescents [28] and adults [29] that examined this question, our results suggest that young adults with higher ACE profiles are especially vulnerable to higher levels of COVID-19 stress. It is unclear as to whether this vulnerability is age-specific or cohort-specific based on the negative findings by Hayden and Salvatore (2022) or whether individuals with high exposure to ACEs are more affected by exposure to proximal stressors across their life course and, in this case, stress associated with COVID-19.

Most stressors associated with COVID-19, especially emotional and relationship stressors, are a direct result of governmental actions such as physical distancing and lockdowns aimed at controlling the spread of the virus to minimize hospital and intensive care unit (ICU) admissions. Interestingly, we found that most of the significant interaction effects between ACEs and COVID-19 on decreases in mental health over time were concentrated in these domains, specifically loneliness and isolation, irritability and frustration, suspiciousness, and distrustfulness of others, and having serious arguments with the people you live with. Financial stressors, while important, may have played a smaller role due to the Canadian government’s income subsidies including the Canadian Emergency Response Benefit (CERB) and extended employment insurance benefits for those who lost their job or had their hours reduced. As the service and hospitality industries which employ a large proportion of young adults were the hardest hit by closures, these employees likely benefited disproportionately from these emergency employment benefits.

It is also possible that some of the observed reductions in mental health may already be occurring for reasons other than the pandemic. Since there could be up to a 2-year lag for some participants between data points, we are unable to discount this possibility. However, those that reported higher COVID-19-related stress had significantly greater increases than those with lower stress and those with both high ACEs and high COVID-19-related stress had the greatest increases in mental health problems. While acknowledging the potential for pre-existing factors that may account for some of these increases over time, these results indicate that those with a combination of higher ACEs and higher COVID-19-related stress had the greatest increases in mental health problems.

Finally, with a universal, public healthcare system in Canada, job losses or significant reductions in hours would not threaten medical coverage and access to care beyond the limitations that were imposed by COVID-19 or the loss of extended employment benefits, such as coverage for dental care, prescriptions, and ancillary health care (e.g., physical therapy). However, there were significant reductions in accessibility to many mental health resources as a result of lockdowns. Most of the mental health care resources that remained available were moved to virtual and phone appointments impeding access for a variety of reasons such as access to appropriate, stable internet services. For example, rural areas, which in countries such as Canada and the US include up to 63% of counties [48], would be more likely to have restricted or nonexistent access to highspeed internet to facilitate online health care.

### Strengths and Limitations

This analysis examined changes in mental health outcomes prospectively from before to during the COVID-19 pandemic among a sample of young adults. Unlike most studies that are either cross-sectional or longitudinal but started after COVID-19 began, we were able to assess these changes using baseline data collected prior to the COVID-19 pandemic and the governmental responses/lockdowns. The study uses a broad array of validated measures of mental health outcomes including depression, anxiety, hostility, and perceived stress that were measured both prior to and during COVID-19.

Notwithstanding these strengths, there were also a number of limitations, First, the COVID-19-related stress measures taken from an international study initiated at the beginning of the pandemic by Lavoie and Bacon [39] have not been evaluated in relation to their psychometric properties. As COVID-19 research continues, there has been little time to assess the psychometrics of measures developed to gauge the impact of the pandemic. Second, of the 248 eligible participants, only 171 (69%) completed the COVID-19 online survey. While this smaller sample may have reduced statistical power to assess moderating effects, a clustering of significant interactions between ACEs and COVID-19 stress over time were identified suggesting the effects are likely quite robust. Third, as the COVID-19 survey was conducted between 4 to 6 months into the pandemic when COVID-19 restrictions were being loosened, tracking changes through later phases of the pandemic and across waves of high infection rates may produce different results. Finally, the study focused on young adults. While this is an important cohort that may be differentially affected by governmental responses to COVID-19, we were unable to compare these findings to older adults or to adolescents. These limitations notwithstanding, the ability to examine the effect of ACEs on mental health outcomes in relation to the COVID-19 pandemic in a prospective, natural experimental design is a novel opportunity to provide further evidence on the robust effect of ACEs on future stress and longer-term health outcomes.

## 5. Conclusions

Young adults who were exposed to greater numbers of ACEs appear to be more vulnerable to the stressful effects of COVID-19 with respect to mental health beyond any independent, additive effects of each one [5,49,50]. As emerging adults are in a life-stage where they are likely to be significantly affected by the pandemic and governmental responses to mitigate the spread of COVID-19, they may be at greater risk for adverse mental health outcomes compared to other cohorts. Research examining whether middle-aged and older adults [29] as well as younger cohorts [28] with higher ACE profiles have not found those with high ACE profiles to be more vulnerable to the adverse effects of COVID-19-related stress. Further longitudinal research is required to identify whether this vulnerability is limited to this specific cohort or whether it continues across their life course.

The findings of this study emphasize the importance of considering exposure to ACEs as an enduring risk factor in the face of more proximal stressors, making these individuals more vulnerable to the negative effects of COVID-19 stress. As such, individuals with high ACE profiles may benefit from intervention programs and resources directed at mental health during times of population-level events. In addition, the findings highlight the serious impact of wide-scale governmental actions and policies meant to control the spread of the pandemic. These imposed, system-wide responses resulted in a unique set of stressors that had a notable effect on the mental health of young adults with higher ACE profiles. Consideration of previous childhood exposures may provide clinicians with further knowledge to both identify and treat those who are at greater risk to adverse mental health outcomes.

## Figures and Tables

**Table 1:** Descriptive statistics of all measures ^1^.

	Pre-COVID-19	During COVID-19	*p*-Value
N	171	171	
Sex (%)			
Male	42.7		
Female	57.3		
Age, years (Mean, SD)	22.1 (1.5)		
Education (%)			
Grade 12 or less	8.8		
High School diploma (or GED)	19.9		
Partial college/training	21.1		
College/university degree	43.9		
Graduate/professional degree	6.4		
ACEs score, (%)			
0	16.4		
1	25.7		
2	23.4		
3	12.3		
4 or more	22.2		
COVID-19 Measures (Mean, SD)			
Emotional			
Lonely and isolated		2.7 (0.96)	
Irritable, frustrated, or angry		2.6 (0.97)	
Suspicious and distrustful of others		2.2 (0.97)	
Lifestyle			
Less physically active		2.4 (1.17)	
Diet has gotten worse		2.1 (1.02)	
Substance Use			
Increased how often/much I drink alcohol		1.7 (0.90)	
Increased how often/much I use recreational drugs		1.5 (0.83)	
Financial			
Job hours cut/lost income		2.4 (1.33)	
Unable to pay rent/mortgage		1.3 (0.79)	
Unable to pay for food		1.3 (0.72)	
Relationship			
Serious arguments with people I live with		1.8 (0.97)	
Serious physical fights with people I live with		1.1 (0.32)	
Mental Health Outcomes (mean, SD) ^1^			
Depression	16.2 (11.1)	17.2 (12.2)	0.200
Anxiety	**16.8 (5.6)**	**17.8 (6.0)**	**0.011**
Hostility	**9.4 (2.6)**	**10.1 (3.0)**	**0.002**
Perceived Stress	27.1 (6.0)	27.4 (6.5)	0.392

^1^ Statistical tests of significance in outcome measures used robust standard errors to adjust for intra-class correlations. Bolded results are significant at *p* < 0.05 (two-tailed).

**Table 2:** Pearson correlations between adverse childhood experiences (ACEs), COVID-19-related stressors, and mental health outcomes during COVID-19 ^1^.

During COVID-19 Mental Health Outcomes					
	ACEs r (*p*-Value)	Depression r (*p*-Value)	Anxiety r (*p*-Value)	Hostility r (*p*-Value)	Perceived Stress r (*p*-Value)
ACEs	-	**0.30 (<0.001)**	**0.34 (<0.001)**	**0.22 (0.004)**	**0.32 (<0.001)**
**COVID-19 Stressors**					
Emotional					
Loneliness	0.14 (0.06)	**0.42 (<0.001)**	**0.24 (0.002)**	**0.20 (0.01)**	**0.38 (<0.001)**
Frustration	**0.18 (0.02)**	**0.41 (<0.001)**	**0.39 (<0.001)**	**0.33 (<0.001)**	**0.46 (<0.001)**
Suspiciousness	0.12 (0.11)	**0.28 (0.002)**	**0.35 (<0.001)**	**0.29 (0.001)**	**0.39 (<0.001)**
Lifestyle					
Physical activity	−0.02 (0.83)	0.05 (0.50)	−0.07 (0.38)	0.04 (0.65)	0.06 (0.45)
Diet	0.00 (0.96)	0.14 (0.08)	0.12 (0.13)	0.14 (0.08)	0.11 (0.16)
Substance Use					
Alcohol use	0.05 (0.55)	0.13 (0.09)	**0.16 (0.03)**	0.15 (0.06)	0.10 (0.19)
Drug use	**0.17 (0.03)**	**0.18 (0.02)**	**0.21 (0.01)**	0.14 (0.06)	**0.19 (0.01)**
Financial					
Income	0.07 (0.34)	**0.17 (0.03)**	0.08 (0.32)	0.08 (0.29)	0.14 (0.07)
Rent/mortgage	**0.33 (<0.001)**	**0.22 (0.01)**	0.12 (0.12)	**0.16 (0.04)**	**0.23 (0.003)**
Food	**0.33 (<0.001)**	**0.33 (<0.001)**	**0.18 (0.02)**	**0.18 (0.02)**	**0.24 (0.002)**
Relationship					
Arguments	**0.20 (0.01)**	**0.37 (<0.001)**	**0.29 (<0.001)**	**0.34 (<0.001)**	**0.37 (<0.001)**
Physical fights	0.10 (0.20)	0.06 (0.46)	−0.05 (0.56)	0.07 (0.40)	0.02 (0.85)

^1^ Bolded results are significant at *p* < 0.05 (two-tailed).

**Table 3:** Longitudinal mixed effects models predicting changes in mental health outcomes as a result of the interaction between COVID-19 stressors and exposure to ACEs over time ^1^.

COVID-19-Related Stress∗ACEs∗Time Regression Coefficients	Mental Health Outcomes			
	Depression *Estimate* (*p*-Value)	Anxiety *Estimate* (*p*-Value)	Hostility *Estimate* (*p*-Value)	Perceived Stress *Estimate* (*p*-Value)
Emotional				
Loneliness	**1.36 (0.02) ^†‡^**	**0.94 (0.002)**	**0.48 (0.002) ^†^**	**0.79 (0.01) ^†^**
Frustration	**1.27 (0.03) ^†‡^**	0.30 (0.32) ^†^	**0.43 (0.004) ^†^**	**0.83 (0.01) ^†^**
Suspiciousness	0.62 (0.27)	**0.70 (0.02) ^†^**	**0.29 (<0.05) ^†^**	**0.69 (0.03) ^†^**
Lifestyle				
Physical activity	−0.14 (0.76)	0.00 (0.99)	**0.30 (0.01) ^†^**	−0.16 (0.53)
Diet	0.65 (0.2)	0.17 (0.53)	**0.44 (0.001) ^†^**	0.18 (0.52)
Substance Use				
Alcohol use	1.04 (0.14)	−0.25 (0.51)	−0.08 (0.68)	**1.18 (0.003)**
Drug use	1.34 (0.07)	0.31 (0.44)	0.11 (0.59)	−0.13 (0.76)
Financial				
Income	0.28 (0.51) ^†‡^	0.15 (0.51)	**0.26 (0.02) ^†^**	0.34 (0.14) ^†^
Rent/mortgage	−0.08 (0.9) ^‡^	0.43 (0.19)	0.25 (0.15) ^†^	0.17 (0.64) ^†^
Food	−0.47 (0.52) ^†‡^	0.55 (0.14)	**0.40 (0.04) ^†^**	0.06 (0.88)
Relationship				
Arguments	−0.30 (0.57) ^†‡^	**0.59 (0.03) ^†^**	**0.52 (<0.001) ^†^**	−0.03 (0.93) ^†^
Physical fights	0.52 (0.75)	0.84 (0.32)	0.17 (0.71)	1.41 (0.13)

^†^ indicates significant independent two-way interaction between COVID-19-stressors and time. **^‡^** indicates significant independent two-way interaction between ACEs and time. ^1^ The three-way interaction regression coefficients are presented for illustrative purposes. The full models with all main-effect and two- and three-way interaction regression coefficients are presented in Appendix A, Table A1 and Table A2. All models are adjusted for sex, age, and education status at pre-COVID-19 baseline. Bold results are significant at *p* < 0.05 (two-tailed).

## Data Availability

The data presented in this study may be made available upon request from the corresponding author.

## Appendix A

**Table A1:** Two-way Longitudinal Mixed Effects Regression Models Predicting Changes in Mental Health Outcomes by COVID-19 Proximal Stressors and Exposure to ACEs with Time.

Two-Way Mixed Effects Models	Mental Health Outcomes			
	Depression *Estimate* (*p*-Value)	Anxiety *Estimate* (*p*-Value)	Hostility *Estimate* (*p*-Value)	Perceived Stress *Estimate* (*p*-Value)
**Emotional**				
Loneliness				
Intercept	2.00 (0.87)	**23.60 (<0.001)**	**7.96 (0.01)**	**23.51 (<0.001)**
Loneliness	−1.38 (0.49)	−0.71 (0.52)	*−1.01 (0.06)*	−0.87 (0.46)
ACEs	**5.39 (<0.001)**	0.59 (0.43)	0.55 (0.14)	1.29 (0.11)
Time	−2.51 (0.30)	−1.10 (0.38)	−0.73 (0.26)	*−2.24 (0.10)*
Loneliness ∗ Time	**2.10 (0.01)**	0.65 (0.12)	**0.51 (0.02)**	**1.004 (0.03)**
ACEs ∗ Time	**−1.11 (0.04)**	0.19 (0.52)	−0.00 (0.99)	−0.06 (0.84)
Frustration				
Intercept	0.62 (0.96)	**25.15 (<0.001)**	**9.44 (0.002)**	**25.39 (<0.001)**
Frustration	−1.56 (0.43)	−1.37 (0.21)	**−1.64 (0.002)**	−1.76 (0.13)
ACEs	**5.76 (<0.001)**	0.75 (0.33)	*0.65 (0.08)*	*1.55 (0.06)*
Time	−2.2 (0.34)	*−2.33 (0.06)*	**−1.54 (0.01)**	**−3.28 (0.01)**
Frustration ∗ Time	**2.07 (0.01)**	**1.17 (0.01)**	**0.86 (<0.001)**	**1.47 (0.001)**
ACEs ∗ Time	**−1.17 (0.03)**	0.14 (0.63)	−0.04 (0.79)	−0.14 (0.66)
Suspiciousness				
Intercept	−12.99 (0.27)	**22.22 (<0.001)**	*5.85 (0.05)*	**18.97 (0.003)**
Suspiciousness	2.34 (0.23)	−1.29 (0.23)	−0.56 (0.29)	−0.43 (0.71)
ACEs	**5.16 (<0.001)**	0.56 (0.45)	0.48 (0.20)	1.21 (0.13)
Time	2.04 (0.35)	*−1.81 (0.10)*	−0.45 (0.43)	−1.75 (0.14)
Suspiciousness ∗ Time	0.36 (0.65)	**1.09 (0.01)**	**0.47 (0.02)**	**0.96 (0.03)**
ACEs ∗ Time	*−0.92 (0.10)*	0.22 (0.44)	0.03 (0.83)	0.01 (0.99)
**Physical**				
Physical Inactivity				
Intercept	−4.83 (0.69)	**21.94 (0.001)**	**8.17 (0.01)**	**19.6 (0.003)**
Physical Inactivity	0.86 (0.6)	0.02 (0.99)	**−1.22 (0.01)**	0.12 (0.9)
ACEs	**5.00 (<0.001)**	0.49 (0.52)	0.52 (0.16)	1.26 (0.12)
Time	2.42 (0.23)	−0.01 (0.99)	−0.62 (0.24)	0.28 (0.80)
Physical Inactivity ∗ Time	0.06 (0.92)	0.22 (0.52)	**0.51 (0.004)**	0.07 (0.85)
ACEs ∗ Time	−0.86 (0.12)	0.24 (0.4)	0.02 (0.90)	−0.01 (0.99)
Poor Diet				
Intercept	−4.27 (0.72)	**23.41 (<0.001)**	**7.7 (0.01)**	**21.93 (0.001)**
Poor Diet	0.80 (0.65)	−0.77 (0.43)	**−1.33 (0.01)**	−1.09 (0.28)
ACEs	**5.01 (0.001)**	0.59 (0.45)	*0.66 (0.08)*	*1.44 (0.08)*
Time	2.25 (0.23)	−0.48 (0.62)	−0.42 (0.39)	−0.67 (0.52)
Poor Diet ∗ Time	0.18 (0.80)	0.53 (0.15)	**0.53 (0.01)**	0.61 (0.13)
ACEs ∗ Time	−0.90 (0.11)	0.18 (0.55)	−0.03 (0.83)	−0.09 (0.78)
**Substance Use**				
Alcohol use				
Intercept	−8.50 (0.48)	**19.01 (0.003)**	**6.13 (0.04)**	**20.64 (0.001)**
Increased Alcohol	1.42 (0.51)	0.89 (0.45)	−0.67 (0.25)	−0.83 (0.50)
ACEs	**5.4 (<0.001)**	0.52 (0.49)	0.50 (0.18)	*1.32 (0.10)*
Time	*3.45 (0.08)*	1.33 (0.19)	0.19 (0.72)	−0.20 (0.86)
Increased Alcohol ∗ Time	−0.36 (0.67)	−0.50 (0.27)	0.24 (0.30)	0.33 (0.50)
ACEs ∗ Time	*−0.97 (0.08)*	0.26 (0.38)	0.03 (0.82)	−0.01 (0.98)
Increased Drug Use				
Intercept	−8.44 (0.48)	**19.46 (0.002)**	*5.95 (0.05)*	**16.48 (0.01)**
Increased Drug Use	1.69 (0.47)	1.43 (0.27)	−0.22 (0.73)	0.85 (0.53)
ACEs	**5.21 (<0.001)**	0.40 (0.61)	0.44 (0.25)	*1.40 (0.09)*
Time	2.57 (0.17)	0.81 (0.42)	0.17 (0.73)	0.58 (0.58)
Increased Drugs ∗ Time	−0.10 (0.92)	−0.30 (0.55)	0.19 (0.45)	−0.15 (0.78)
ACEs ∗ Time	−0.84 (0.14)	0.31 (0.31)	0.07 (0.66)	0.00 (0.99)
**Financial**				
Decreased Income				
Intercept	1.55 (0.90)	**20.84 (0.001)**	**7.16 (0.02)**	**24.83 (<0.001)**
Decreased Income	−2.36 (0.11)	0.25 (0.76)	**−1.05 (0.01)**	**−2.22 (0.01)**
ACEs	**5.75 (<0.001)**	0.46 (0.56)	0.61 (0.11)	*1.54 (0.06)*
Time	0.33 (0.86)	0.56 (0.57)	−0.27 (0.58)	−1.61 (0.12)
Decreased Income ∗ Time	**1.21 (0.04)**	−0.04 (0.9)	**0.39 (0.01)**	**0.89 (0.01)**
ACEs ∗ Time	**−1.12 (0.048)**	0.28 (0.35)	0.00 (0.99)	−0.09 (0.77)
Inability to Pay Rent/mortgage				
Intercept	−3.70 (0.76)	**19.61 (0.001)**	*6.04 (0.05)*	**21.16 (0.001)**
Rent/Mortgage	−1.02 (0.69)	−1.20 (0.38)	*−1.35 (0.05)*	*−2.53 (0.09)*
ACEs	**5.85 (<0.001)**	1.00 (0.18)	*0.64 (0.10)*	**1.71 (0.04)**
Time	1.93 (0.26)	0.26 (0.77)	−0.07 (0.88)	−0.94 (0.33)
Rent/Mortgage ∗ Time	1.12 (0.27)	0.54 (0.31)	**0.57 (0.04)**	**1.27 (0.03)**
ACEs ∗ Time	**−1.29 (0.03)**	0.07 (0.82)	−0.03 (0.84)	−0.21 (0.52)
Inability to Pay for Food				
Intercept	*−0.06 (1.00)*	**19.78 (0.001)**	**6.15 (0.04)**	**22.07 (0.001)**
Food	−3.16 (0.25)	−1.28 (0.38)	**−1.91 (0.01)**	−2.27 (0.16)
ACEs	**5.84 (<0.001)**	0.87 (0.24)	*0.76 (0.05)*	*1.58 (0.06)*
Time	0.37 (0.83)	0.01 (0.99)	−0.23 (0.62)	−0.86 (0.38)
Food ∗ Time	**2.53 (0.02)**	0.70 (0.21)	**0.78 (0.01)**	*1.18 (0.06)*
ACEs ∗ Time	**−1.35 (0.02)**	0.09 (0.76)	−0.07 (0.62)	−0.17 (0.61)
**Relationship**				
Arguments				
Intercept	−9.43 (0.43)	**18.3 (0.004)**	**6.7 (0.03)**	**16.4 (0.01)**
Serious argument	−2.54 (0.21)	−1.45 (0.17)	**−1.79 (0.001)**	−0.92 (0.44)
ACEs	**5.69 (<0.001)**	0.88 (0.23)	*0.73 (0.05)*	*1.36 (0.10)*
Time	−0.49 (0.78)	−0.74 (0.41)	*−0.84 (0.07)*	−1.2 (0.24)
Serious argument ∗ Time	**2.31 (0.004)**	**0.98 (0.02)**	**0.92 (<0.001)**	**1.03 (0.03)**
ACEs ∗ Time	**−1.30 (0.02)**	0.05 (0.85)	−0.09 (0.53)	−0.13 (0.68)
Physical fights				
Intercept	1.01 (0.94)	**19.23 (0.01)**	5.44 (0.11)	**20.59 (0.01)**
Physical Fight	−6.72 (0.26)	−0.24 (0.94)	−0.70 (0.67)	−1.82 (0.60)
ACEs	**5.42 (<0.001)**	0.68 (0.36)	0.48 (0.20)	*1.33 (0.10)*
Time	0.10 (0.97)	1.20 (0.41)	0.15 (0.84)	0.02 (0.99)
Physical Fight ∗ Time	2.60 (0.28)	−0.45 (0.72)	0.37 (0.57)	0.42 (0.76)
ACEs ∗ Time	*−1.01 (0.07)*	0.20 (0.49)	0.04 (0.81)	−0.02 (0.94)

All models are adjusted for sex, age, and education status at pre COVID-19. Bold results are significant at *p* < 0.05 (two-tailed), *Italicized* results are at *p* 0.05–0.1 (two-tailed).

**Table A2:** Three-way Longitudinal Mixed Effects Models Predicting Changes in Mental Health Outcomes as a Result of The Interaction between COVID-19 Proximal Stressors and Exposure to ACEs Over Time.

**Three-Way** **Mixed Effects Models**	**Mental Health Outcomes**			
	**Depression** ***Estimate* (*p*-** **Value)**	**Anxiety** ***Estimate* (*p*-** **Value)**	**Hostility ** ***Estimate* (*p*-** **Value)**	**Perceived Stress ** ***Estimate* (*p*-** **Value)**
**Emotional**				
Loneliness				
Intercept	−7.11 (0.61)	*14.76 (0.05)*	2.86 (0.44)	**16.13 (0.04)**
Loneliness	1.85 (0.6)	2.43 (0.21)	0.92 (0.33)	1.81 (0.38)
Time	4.78 (0.22)	*3.9 (0.05)*	*1.82 (0.07)*	2.01 (0.36)
ACEs	**9.8 (0.02)**	**4.87 (0.04)**	**3.17 (0.005)**	*4.92 (0.05)*
Time ∗ ACEs	**−4.85 (0.004)**	**−2.38 (0.01)**	**−1.31 (0.003)**	**−2.24 (0.02)**
Time ∗ Loneliness	−0.65 (0.64)	*−1.24 (0.09)*	−0.45 (0.22)	−0.6 (0.45)
Loneliness ∗ ACEs	−1.6 (0.27)	*−1.56 (0.05)*	**−0.96 (0.01)**	−1.32 (0.12)
Loneliness ∗ ACEs ∗ Time	**1.36 (0.02)**	**0.94 (0.002)**	**0.48 (0.002)**	**0.79 (0.01)**
Frustration				
Intercept	−8.88 (0.53)	**22.5 (0.003)**	4.94 (0.17)	**17.06 (0.03)**
Frustration	1.87 (0.6)	−0.44 (0.82)	0.07 (0.94)	1.31 (0.52)
Time	4.53 (0.23)	−0.76 (0.7)	0.71 (0.47)	1.14 (0.59)
ACEs	**10.43 (0.01)**	2.03 (0.38)	**2.98 (0.01)**	**5.72 (0.02)**
Time ∗ ACEs	**−4.63 (0.01)**	−0.67 (0.44)	**−1.2 (0.01)**	**−2.41 (0.01)**
Time ∗ Frustration	−0.49 (0.72)	0.57 (0.43)	0.01 (0.99)	−0.2 (0.79)
Frustration ∗ ACEs	−1.72 (0.24)	−0.47 (0.56)	**−0.86 (0.03)**	*−1.53 (0.07)*
Frustration ∗ ACEs ∗ Time	**1.27 (0.03)**	0.3 (0.32)	**0.43 (0.004)**	**0.83 (0.01)**
Suspiciousness				
Intercept	−17.48 (0.2)	*13.7 (0.06)*	2.57 (0.47)	11.02 (0.14)
Suspiciousness	4.35 (0.24)	2.58 (0.2)	0.93 (0.34)	3.15 (0.14)
Time	5.1 (0.15)	1.56 (0.38)	0.97 (0.29)	1.65 (0.4)
ACEs	**7.2 (0.04)**	**4.53 (0.02)**	**2.01 (0.03)**	**4.85 (0.02)**
Time ∗ACEs	*−2.33 (0.1)*	*−1.34 (0.06)*	*−0.63 (0.09)*	**−1.55 (0.04)**
Time ∗ Suspiciousness	−1.03 (0.49)	−0.43 (0.57)	−0.17 (0.66)	−0.57 (0.48)
Suspiciousness ∗ ACEs	−0.91 (0.52)	**−1.77 (0.02)**	*−0.68 (0.08)*	*−1.61 (0.05)*
Suspiciousness ∗ ACEs ∗ Time	0.62 (0.27)	**0.7 (0.02)**	*0.29 (0.05)*	**0.69 (0.03)**
**Physical**				
Physical Inactivity				
Intercept	−0.47 (0.97)	**22.95 (0.001)**	4.77 (0.15)	**22.35 (0.002)**
Physical Inactivity	−1.81 (0.53)	−0.71 (0.66)	0.21 (0.78)	−1.31 (0.43)
Time	1.74 (0.56)	0.01 (1)	0.82 (0.29)	−0.51 (0.76)
ACEs	1.84 (0.56)	−0.38 (0.83)	**2.24 (0.01)**	−0.43 (0.81)
Time ∗ ACEs	−0.51 (0.68)	0.24 (0.72)	**−0.72 (0.03)**	0.39 (0.58)
Time ∗ Physical Inactivity	0.35 (0.76)	0.21 (0.73)	−0.1 (0.74)	0.41 (0.53)
Physical Inactivity ∗ ACEs	1.31 (0.26)	0.36 (0.58)	**−0.71 (0.02)**	0.7 (0.3)
Physical Inactivity ∗ ACEs ∗ Time	−0.14 (0.76)	0 (0.99)	**0.3 (0.01)**	−0.16 (0.53)
Poor Diet				
Intercept	−8.51 (0.51)	**22.9 (0.001)**	3.24 (0.32)	**20.89 (0.004)**
Poor Diet	1.02 (0.76)	−1.67 (0.36)	0.77 (0.37)	−1.33 (0.49)
Time	*5.14 (0.08)*	0.28 (0.85)	**1.57 (0.04)**	0.16 (0.92)
ACEs	*5.21 (0.09)*	−0.32 (0.85)	**2.73 (0.001)**	1.21 (0.5)
Time ∗ ACEs	*−2.29 (0.06)*	−0.18 (0.78)	**−0.99 (0.002)**	−0.49 (0.48)
Time ∗ Poor Diet	−1.25 (0.35)	0.15 (0.82)	−0.45 (0.19)	0.2 (0.79)
Poor Diet ∗ ACEs	−0.1 (0.94)	0.41 (0.56)	**−0.96 (0.004)**	0.11 (0.89)
Poor Diet ∗ ACEs ∗ Time	0.65 (0.2)	0.17 (0.53)	**0.44 (0.001)**	0.18 (0.52)
**Substance Use**				
Alcohol use				
Intercept	−11.72 (0.38)	**21.63 (0.002)**	*6.21 (0.07)*	*12.86 (0.08)*
Increased Alcohol	4.28 (0.3)	−0.71 (0.76)	−0.8 (0.48)	*4.43 (0.06)*
Time	**6.69 (0.02)**	0.6 (0.69)	−0.07 (0.93)	**3.48 (0.03)**
ACEs	**7.59 (0.01)**	−0.74 (0.67)	0.4 (0.63)	**5.36 (0.003)**
Time ∗ ACEs	**−2.6 (0.04)**	0.64 (0.33)	0.16 (0.63)	**−1.85 (0.01)**
Time ∗ Increased Alcohol	−2.48 (0.14)	−0.02 (0.98)	0.41 (0.37)	**−2.07 (0.02)**
Increased Alcohol ∗ ACEs	−1.41 (0.42)	0.82 (0.42)	0.06 (0.9)	**−2.59 (0.01)**
Increased Alcohol ∗ ACEs ∗ Time	1.04 (0.14)	−0.25 (0.51)	−0.08 (0.68)	**1.18 (0.003)**
Increased Drug Use				
Intercept	−16.65 (0.21)	**16.98 (0.02)**	4.95 (0.14)	**18.06 (0.01)**
Increased Drugs	7.72 (0.11)	3.28 (0.22)	0.52 (0.69)	−0.35 (0.9)
Time	**6.73 (0.02)**	1.75 (0.27)	0.51 (0.52)	0.18 (0.92)
ACEs	**8.91 (0.003)**	1.53 (0.35)	0.9 (0.27)	0.66 (0.7)
Time ∗ ACEs	**−2.72 (0.02)**	−0.12 (0.85)	−0.08 (0.79)	0.19 (0.78)
Time ∗ Increased Drugs	−3.16 (0.1)	−1 (0.33)	−0.06 (0.92)	0.15 (0.89)
Increased Drugs ∗ ACEs	−2.63 (0.15)	−0.81 (0.43)	−0.33 (0.52)	0.52 (0.62)
Increased Drugs ∗ ACEs ∗ Time	*1.34 (0.07)*	0.31 (0.44)	0.11 (0.59)	−0.13 (0.76)
**Financial**				
Decreased Income				
Intercept	2.48 (0.85)	**19.43 (0.01)**	3.82 (0.24)	**21.8 (0.002)**
Decreased Income	−2.47 (0.34)	0.89 (0.53)	0.37 (0.58)	−0.83 (0.57)
Time	1.59 (0.55)	1.22 (0.39)	0.9 (0.2)	−0.05 (0.97)
ACEs	*5.62 (0.05)*	1.21 (0.44)	**2.29 (0.003)**	*3.18 (0.05)*
Time ∗ ACEs	−1.78 (0.12)	−0.07 (0.91)	**−0.62 (0.04)**	−0.91 (0.15)
Time ∗ Decreased Income	0.65 (0.53)	−0.33 (0.54)	−0.13 (0.62)	0.2 (0.73)
Decreased Income ∗ ACEs	0.05 (0.96)	−0.32 (0.58)	**−0.71 (0.01)**	−0.69 (0.24)
Decreased Income ∗ ACEs ∗ Time	0.28 (0.51)	0.15 (0.51)	**0.26 (0.02)**	0.34 (0.14)
Inability to Pay Rent/mortgage				
Intercept	3.85 (0.77)	**18.79 (0.01)**	5.17 (0.12)	**23.89 (0.001)**
Rent/Mortgage	−5.42 (0.24)	−0.05 (0.98)	−0.44 (0.72)	−3.78 (0.16)
Time	1.68 (0.52)	1.62 (0.23)	0.72 (0.31)	−0.41 (0.78)
ACEs	3.26 (0.22)	1.68 (0.24)	1.17 (0.11)	0.97 (0.53)
Time ∗ ACEs	−1.17 (0.28)	−0.55 (0.32)	−0.39 (0.18)	−0.45 (0.46)
Time ∗ Rent/Mortgage	1.32 (0.48)	−0.51 (0.59)	−0.04 (0.93)	0.86 (0.41)
Rent/Mortgage ∗ ACEs	1.8 (0.25)	−0.47 (0.58)	−0.38 (0.39)	0.51 (0.57)
Rent/Mortgage ∗ ACEs ∗ Time	−0.08 (0.9)	0.43 (0.19)	0.25 (0.15)	0.17 (0.64)
Inability to Pay for Food				
Intercept	11.26 (0.39)	**18.12 (0.01)**	3.02 (0.37)	**25.99 (0.001)**
Food	*−10.83 (0.05)*	0.6 (0.84)	0.59 (0.69)	−4.55 (0.16)
Time	−1.19 (0.69)	1.81 (0.23)	1.08 (0.17)	−0.66 (0.7)
ACEs	1.92 (0.5)	1.83 (0.22)	**2.04 (0.01)**	0.41 (0.8)
Time ∗ACEs	−0.72 (0.53)	−0.65 (0.26)	**−0.62 (0.04)**	−0.25 (0.7)
Time ∗ Food	*3.78 (0.09)*	−0.75 (0.51)	−0.28 (0.63)	1.02 (0.42)
Food ∗ ACEs	2.88 (0.11)	−0.71 (0.46)	*−0.95 (0.05)*	0.85 (0.42)
Food ∗ ACEs ∗ Time	−0.47 (0.52)	0.55 (0.14)	**0.40 (0.04)**	0.06 (0.88)
**Relationship**				
Arguments				
Intercept	−4.16 (0.75)	**14.19 (0.03)**	2.35 (0.47)	**17.58 (0.01)**
Serious argument	*−6.37 (0.07)*	0.68 (0.71)	0.75 (0.4)	−1.85 (0.37)
Time	−1.6 (0.54)	1.39 (0.3)	1.04 (0.11)	−1.29 (0.39)
ACEs	2.35 (0.41)	*2.71 (0.07)*	**2.93 (<0.001)**	0.54 (0.74)
Time ∗ ACEs	−0.74 (0.51)	*−1.03 (0.07)*	**−1.04 (<0.001)**	−0.09 (0.89)
Time ∗ Serious argument	**2.96 (0.03)**	−0.27 (0.7)	−0.18 (0.61)	1.08 (0.17)
Serious argument ∗ ACEs	1.8 (0.18)	−1 (0.15)	**−1.2 (0.001)**	0.44 (0.58)
Serious argument ∗ ACEs ∗ Time	−0.3 (0.57)	**0.59 (0.03)**	**0.52 (<0.001)**	−0.03 (0.93)
Physical fights				
Intercept	−6.48 (0.73)	12.26 (0.2)	4.58 (0.34)	7.24 (0.48)
Physical Fight	−0.73 (0.95)	6.01 (0.37)	0.19 (0.96)	9.77 (0.18)
Time	1.58 (0.77)	3.6 (0.2)	0.63 (0.67)	4.05 (0.19)
ACEs	*7.8 (0.09)*	3.17 (0.2)	0.83 (0.51)	**5.94 (0.03)**
Time ∗ ACEs	−1.57 (0.4)	−0.71 (0.46)	−0.14 (0.78)	−1.54 (0.15)
Time ∗ Physical Fight	1.2 (0.81)	−2.72 (0.3)	−0.08 (0.95)	−3.39 (0.24)
Physical Fight ∗ ACEs	−2.21 (0.59)	−2.31 (0.29)	−0.33 (0.77)	*−4.28 (0.07)*
Physical Fight ∗ ACEs ∗ Time	0.52 (0.75)	0.84 (0.32)	0.17 (0.71)	1.41 (0.13)

All models are adjusted for sex, age, and education status at pre COVID-19. Bold results are significant at *p* < 0.05 (two-tailed), *Italicized* results are at *p* 0.05–0.1 (two-tailed).
